# Supplementary material for: From sequence to enzyme mechanism using multi-label machine learning
Source: BMC Bioinformatics. 2014 May 19;15:150. doi: 10.1186/1471-2105-15-150 (PMC4229970; doi:10.1186/1471-2105-15-150)
Supplement: Additional file 2 — Java code of ml2db. Additional file ml2db_code.tar.gz contains the Java source code to run the multi-label machine learning experiments and save the results to database. The code’s Javadoc is included. [file 1471-2105-15-150-S2.zip › additional file 2/ml2db/ml2db/doc/main/ml2db/mulan/learn/evaluation/package-summary.html]

main.ml2db.mulan.learn.evaluation


JavaScript is disabled on your browser.


- Overview
- Package
- Class
- Use
- Tree
- Deprecated
- Index
- Help

- Prev Package
- Next Package

- Frames
- No Frames

- All Classes

# Package main.ml2db.mulan.learn.evaluation

- Class Summary

  | Class | Description |
  | --- | --- |
  |  |  |
  | --- | --- |
  | EvaluationParameters | Contains the evaluation parameters (micro-macro precision, micro-macro accuracy etc.) of a Multi label Mulan experiment. |
  | EvaluatorNoAUC | Prepares the evaluation measures taking into consideration the dataset size. |
  | FalseNegatives | Class |
  | FalsePositives | Class |
  | MicroFMeasurePrinter | A micro f measure version that also prints out the number of true positives, false positives and false negatives |
  | TruePositives | Class |

- Overview
- Package
- Class
- Use
- Tree
- Deprecated
- Index
- Help

- Prev Package
- Next Package

- Frames
- No Frames

- All Classes
